# Hyperplasia and hypertrophy of pulmonary neuroepithelial bodies, presumed airway hypoxia sensors, in hypoxia-inducible factor prolyl hydroxylase-deficient mice

**DOI:** 10.2147/HP.S103957

**Published:** 2016-04-12

**Authors:** Jie Pan, Tammie Bishop, Peter J Ratcliffe, Herman Yeger, Ernest Cutz

**Affiliations:** 1 Division of Pathology, Department of Pediatric Laboratory Medicine, The Research Institute, The Hospital for Sick Children; 2 Department of Laboratory Medicine and Pathobiology, University of Toronto, Toronto, ON, Canada; 3 Nuffield Department of Medicine, Henry Wellcome Building for Molecular Physiology, University of Oxford, Oxford, UK

**Keywords:** hypoxia, PHD, HIF, Mash1, neuroendocrine, pulmonary

## Abstract

Pulmonary neuroepithelial bodies (NEBs), presumed polymodal airway sensors, consist of innervated clusters of amine (serotonin) and peptide-producing cells. While NEB responses to acute hypoxia are mediated by a membrane-bound O_2_ sensor complex, responses to sustained and/or chronic hypoxia involve a prolyl hydroxylase (PHD)–hypoxia-inducible factor-dependent mechanism. We have previously reported hyperplasia of NEBs in the lungs of *Phd1*−/− mice associated with enhanced serotonin secretion. Here we use a novel multilabel immunofluorescence method to assess NEB distribution, frequency, and size, together with the number and size of NEB cell nuclei, and to colocalize multiple cytoplasmic and nuclear epitopes in the lungs of *Phd1*−/−, *Phd2*+/−, and *Phd3*−/− mice and compare them with wild-type controls. To define the mechanisms of NEB cell hyperplasia, we used antibodies against Mash1 and Prox1 (neurogenic genes involved in NEB cell differentiation/maturation), hypoxia-inducible factor-1alpha, and the cell proliferation marker Ki67. Morphometric analysis of (% total lung area) immunostaining for synaptophysin (% synaptophysin), a cytoplasmic marker of NEB cells, was significantly increased in *Phd1*−/− and *Phd3*−/− mice compared to wild-type mice. In addition, NEB size and the number and size of NEB nuclei were also significantly increased, indicating that deficiency of *Phd*s is associated with striking hyperplasia and hypertrophy of NEBs. In *Phd2*+/− mice, while mean % synaptophysin was comparable to wild-type controls, the NEB size was moderately increased, suggesting an effect even in heterozygotes. NEBs in all *Phd*-deficient mice showed increased expression of Mash1, Prox1, Ki67, and hypoxia-inducible factor-1alpha, in keeping with enhanced differentiation from precursor cells and a minor component of cell proliferation. Since the loss of PHD activity mimics chronic hypoxia, our data provide critical information on the potential role of PHDs in the pathobiology and mechanisms of NEB cell hyperplasia that is relevant to a number of pediatric lung disorders.

## Introduction

The pulmonary neuroendocrine cell (PNEC) system is comprised of solitary cells and distinctive innervated clusters called neuroepithelial bodies (NEBs), which are localized within the airway mucosa of mammalian lungs. PNEC/NEB cells produce bioactive amines (serotonin [5-HT]) and a variety of peptides (bombesin in humans, CGRP in rodents) that have local or distant effects.^1^–^3^ The precise function of PNEC/NEB cells is currently unknown although recent studies report a potential role in pulmonary inflammation.^4^ While solitary PNECs are thought to mediate local airway responses, NEBs are presumed to be polymodal airway sensors analogous to carotid body (CB) glomus cells and a part of the body’s O_2_ sensing homeostatic system.^5^,^6^ The evidence supporting their role as airway O_2_ sensors includes: 1) structural features, including localization near airway branch points, an apical surface in contact with airway lumen, and extensive innervation by vagal afferent fibers; 2) expression of a membrane-bound O_2_ sensing molecular complex composed of the multicomponent nicotinamide adenine dinucleotide phosphate (NADPH) oxidase coupled to an O_2_ sensitive K^+^ channel^7^; 3) the observation that exposure to acute hypoxia leads to activation of the O_2_ sensor via Ca^2+^-mediated, dose-dependent 5-HT release^8^; and 4) the observation that 5-HT and other NEB cell-derived neurotransmitters (ie, ATP) activate their respective postsynaptic receptors on vagal afferents, thus transmitting the signals to the brain stem and affecting the control of respiration.^3^ Earlier studies have shown that exposure to chronic, sustained hypoxia in both human and animal models leads to hyperplasia of NEBs similar to CB glomus cells.^9^ We and others have previously reported striking hyperplasia of PNEC/NEBs in several pediatric lung disorders, including chronic lung disease of prematurity (bronchopulmonary dysplasia), sudden infant death syndrome, and neuroendocrine cell hyperplasia of infancy.^1^,^2^ Chronic hypoxia likely plays an important role in the pathobiology of these disorders, although the precise mechanisms are not known. We have recently described marked hyperplasia of NEBs in prolyl hydroxylase 1 (*Phd1*) deficient mice, with a two- to threefold increase in the number and size of NEBs.^10^ Furthermore, we have shown that hyperplastic NEBs in *Phd1*−/− mice exhibit enhanced hypoxia-induced 5-HT secretion compared to wild-type (WT) controls suggesting altered NEB cell function.^11^ Thus, *Phd1*−/− mice may be a useful animal model to investigate the mechanisms and functional consequences of NEB cell hyperplasia. Since PHDs are the principal cellular O_2_ sensors involved in the signal transduction of chronic/sustained hypoxia,^12^,^13^ in the present study, we have performed detailed immunohistochemical and morphometric analysis of NEBs in the lungs of *Phd1*−/−, *Phd2*+/−, and *Phd3*−/− mice, and compared them with WT controls.

To study the potential mechanisms of NEB cell hyperplasia, we used a novel immunohistochemical method applied to formalin-fixed paraffin sections allowing multilabel immunofluorescence with simultaneous detection of cytoplasmic and nuclear epitopes on the same sections.^14^ Using this method, we examined the expression of an NEB cell cytoplasmic neuroendocrine cell marker, synaptophysin (SYP), together with nuclear immunolocalization of neurogenic genes: mammalian achete–scute homolog (Mash1), prospero homeobox 1 (Prox1), essential for NEB cell differentiation during development and postnatally;^15^–^17^ a proliferation marker Ki67; and hypoxia-inducible factor (HIF)-1alpha, the transcriptional mediator of hypoxia-induced expression of numerous genes involved in O_2_ homeostasis.^18^ Here, we show that NEBs in *Phd1*−/− and *Phd3*−/− mice show marked hyperplasia and hypertrophy. On the other hand, in *Phd2*+/− animals, the number and distribution of NEBs were comparable to WT controls, although their size was moderately increased, suggesting a role for PHD2 that was partially impaired in heterozygotes. In all *Phd* deficient mice, expression of Mash1, Prox1, and Ki67 was significantly increased compared to WT controls suggesting that NEB cell hyperplasia in these models is mediated predominantly by differentiation from precursor cells with a minor component of cell division. In addition, HIF-1alpha expression was also increased and colocalized with Mash1, indicating a hypoxia-driven differentiation process.

## Materials and methods

### Animals

All animal experiments were performed in accordance and with the approval of with the UK Home Office Animals (Scientific Procedures) Act 1986 and Local Ethical Review Procedures (University of Oxford Medical Sciences Division Ethical Review Committee). *Phd1*−/− (n=4) and *Phd3*−/− (n=4) mice were on a mixed Swiss/129/SvEv genetic background, while *Phd2*+/− mice (n=4) were on a pure C57BL/6 genetic background, and an equal number of WT mice were used in all experiments, as previously reported.^19^–^21^ Male mice, ~3 months old and from the same litter, were used. Mice were killed by an overdose of isoflurane and exsanguination followed by removal of the lungs and fixation in 4% paraformaldehyde/phosphate-buffered saline (PBS) prior to transfer into 70% ethanol and processing. The samples were embedded in paraffin and sectioned at 4 μm thickness.

### Multilabel immunofluorescence

For immunolocalization of different cytoplasmic and nuclear epitopes on the same sections, we used a newly developed multilabel immunofluorescence method on formalin-fixed paraffin-embedded tissue sections.^14^

As a first step, paraffin was removed using xylene, then rehydrated with different concentrations of ethanol. Antigen retrieval was performed with high pressure cooking in ethylenediaminetetraacetic acid (EDTA)–borate buffer (1 mM EDTA, 10 mM borax [sodium tetraborate, Sigma-Aldrich Co., St Louis, MO, USA], 10 mM boric acid [Sigma-Aldrich Co.] with 0.001% Proclin 300 [Supelco, Bellefonte, PA, USA]) at pH 9.0. To block nonspecific staining, the slides were incubated for 1 hour at room temperature in 5% bovine serum albumin in PBS, without Ca^2+^ and Mg^2+^, containing 15% goat serum. Incubation with primary antibody was performed overnight at 4°C (Table 1). The immunostained slides were washed three times with PBS for 10 minutes. Secondary antibody incubation was performed at room temperature in darkness for 2 hours and slides were washed three times for 10 minutes in darkness. RedDot2 (Biotium, Hayward, CA, USA) was used at a dilution of 1:1,000 for nuclear counter staining. The sections were mounted overnight with Vectorshield fluorescence mounting medium (Vector Labs, Burlingame, CA, USA).

### Stripping and re-probing of immunostained formalin-fixed, paraffin-embedded tissue sections

The stripping of the previous immunolabeled target and re-probing for a new antigen on the same section was performed according to previously reported procedures.^14^ Briefly, the slides were dipped in 100% xylene for a few seconds to remove the sealant and the coverslip. After three washes (5 minutes each) with PBS, the prewarmed tissue stripping solution (consisting of 0.05% sodium dodecyl sulfate (SDS) [Sigma-Aldrich Co.], 0.001% mercaptoethanol, and 0.001% Tween20 in MilliQ water pH 4–5 at 30°C) was added on each slide and incubated for 5 minutes at room temperature under a ventilation hood. The stripping solution was discarded and followed by five washes in PBS (5 minutes each). The high pH antigen retrieval step using a high pressure cooker was repeated once more as previously described. After washing and blocking, slides were ready for re-probing with different antibodies in same sections.

### Confocal microscopy

Fluorescent confocal images of PNEC/NEB cells immunostained for SYP, Mash1, Prox1, Ki67, and HIF-1alpha in double/triple-immunostained sections were obtained using a Leica confocal laser scanning microscope (model TCS-SPE) and LAS-AF software (Leica Microsystems, Wetzlar, Germany), as previously reported.^22^ The variable excitation wavelengths of the krypton/argon laser were 488 nm for fluorescein isothiocyanate conjugate, 568 nm for Texas Red complex, and 695 nm for Alexa Fluor 680 conjugate/RedDot 2 (nuclear counterstaining). Image processing, including color resolution, color separation, and merging of fields, was carried out using Adobe PhotoShop CS2 software (Adobe Systems Incorporated, San Jose, CA, USA). To visualize these images in different color from before and after the stripping/re-probing protocol, pseudo-color was applied with the channel option of Adobe Photoshop CS3 (Adobe Systems Incorporated).

### Morphometric analysis

To determine the distribution, frequency, size, and number of nuclei in NEBs of *Phd*-deficient mice compared to WT controls, we used computer-assisted morphometric methods as previously reported.^10^,^23^ A minimum of five sections per animal were analyzed. As a measure of NEB frequency, we assessed % of SYP immunoreactive (NEB cell cytoplasmic marker) area per 100 mm^2^ of airway epithelium. NEB size was expressed as the total integrated surface of SYP immunoreactive area, total number of NEB nuclei, and average size of NEB nuclei per integrated area. We also assessed the numbers of Mash1, Prox1, Ki67, and HIF-1alpha immunoreactive nuclei and % colocalization of various nuclear epitopes.

### Statistical analysis

A one-way analysis of variance with repeated measures was used for statistical analysis of *Phd*-deficient and WT mice. One-way analysis of variance tests with repeated measures was also used for comparison of PNEC/NEB number and integrated area and nuclear size. All data were expressed as mean ± standard error of the mean.

## Result

**Figure 1:**
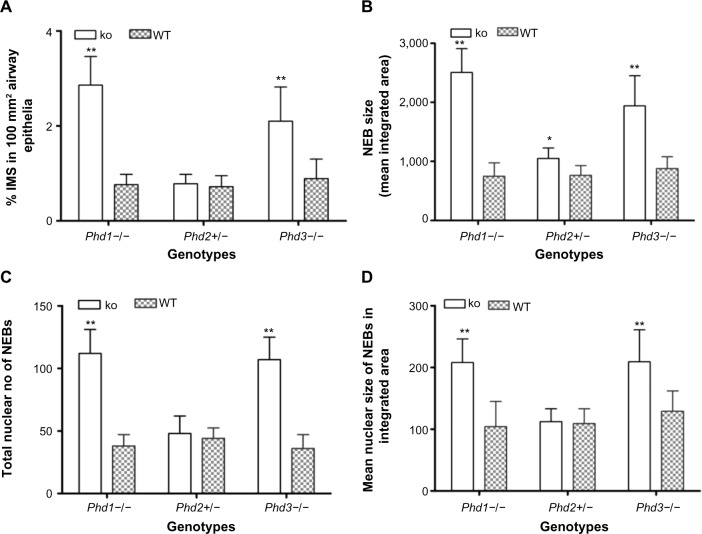
Statistical analysis of morphometric data of neuroepithelial bodies (NEBs) in lungs from *Phd*-deficient mice. **Notes:** (**A**) Comparison of mean frequency of NEBs expressed as % synaptophysin immunoreactive staining (% IMS) area. Values for *Phd1*−/− and *Phd3*−/− mice are significantly increased compared to wild-type (WT) controls (*P*<0.01). No significant difference in NEB frequency is observed in the lungs of *Phd2*+/− mice. (**B**) The mean size of NEBs in both *Phd1*−/− and *Phd3*−/− mice is increased twofold compared to WT control mice (*P*<0.01). In *Phd2*+/− mice, mean size of NEBs is marginally, but significantly, increased compared to WT control mice (*P*<0.05). (**C**) The total number of nuclei in NEBs of *Phd1*−/− and *Phd3*−/− mice is significantly increased compared to WT controls (*P*<0.01). No significant difference is seen between *Phd2*+/− and WT control mice. (**D**) The mean size of NEB cell nuclei is significantly increased (*P*<0.01) in both *Phd1*−/− and *Phd3*−/− mice compared to WT control mice. No significant difference is seen in *Phd2*+/− mice. **P<0.01; *P<0.05. **Abbreviation:** ko, knockout.

The sections of lungs from different *Phd*-deficient genotypes showed unremarkable histology with well-preserved and normally developed airways and alveolar components. The results of morphometric analyses comparing NEBs in the three *Phd*-deficient genotypes and WT controls, including statistical analyses, are shown in Table 2 and Figure 1.

**Figure 2:**
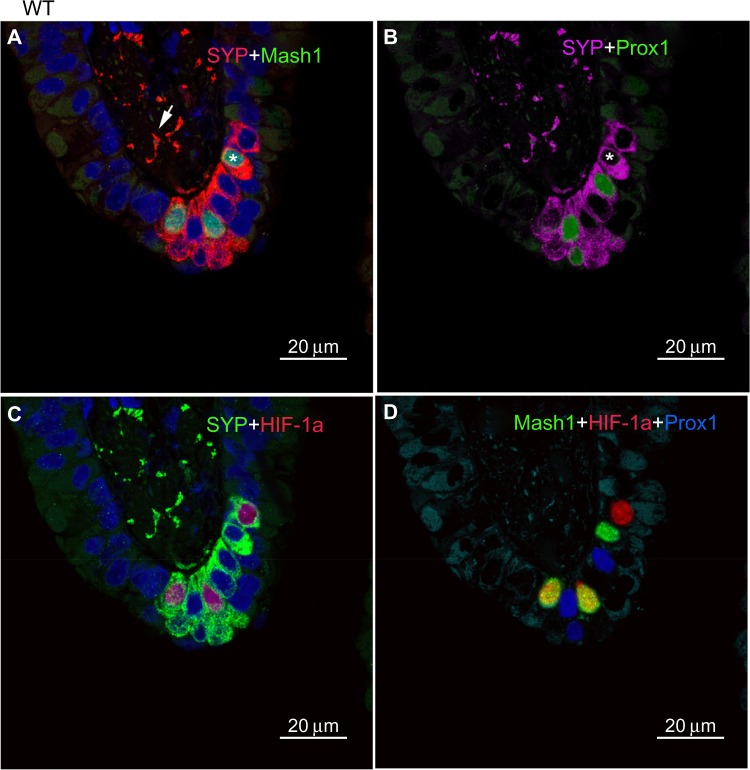
Neuroepithelial bodies (NEBs) in the lungs of wild-type control mice. **Notes:** (**A**) Portion of airway epithelium with NEB immunostained for synaptophysin (SYP, rhodamine, red) and Mash1 (FITC, green). SYP diffusely stains the cytoplasm of NEB cells and nerve fibers (arrow) in the submucosa. Only a few NEB cell nuclei are Mash1-positive (asterisk). (**B**) Same NEB as in (**A**), after antigen stripping and re-probing for Prox1. Prox1 is expressed in Mash1-negative nuclei (asterisk; compare with [**A**]) (scale bar 20 μm). (**C**) Double immunostaining for SYP and hypoxia-inducible factor (HIF)-1alpha in the same NEB as in (**A**) and (**B**). Three nuclei are positive for HIF-1alpha, two coexpress Mash1, and one (upper) expresses each protein alone (compare with [**A**]). (**D**) Merged image of triple immunostaining for Mash1, HIF-1alpha, and Prox1. Two nuclei coexpressing Mash1 and HIF-1alpha (yellow); others show a single nucleus positive for Mash1 (FITC, green), Prox1 (blue pseudo-color), or HIF-1alpha (red). **Abbreviations:** WT, wild-type; FITC, fluorescein isothiocyanate.

In age-matched WT counterparts used as controls for each experimental group, the distribution, frequency, and size of NEBs correspond to previous descriptions.^23^ The overall frequency of NEB (expressed as % SYP immunoreactive area/100 mm^2^, % SYP) ranged between 0.72±0.2 and 0.89±0.4 and an average NEB size expressed as relative integrated surface area varied between 748±227 and 878.2±199 (Table 2). In control lungs, a typical NEB located within the airway epithelium consisted of between five and 15 closely packed cells. Double immunostaining for SYP (a marker of NEB) and Mash1 showed positive nuclei in a few NEB cells with all other airway epithelial cells negative (Figure 2A), confirming the specificity and restriction of Mash1 expression to cells with neuroendocrine features. The same NEB, re-probed with antibody to Prox1, showed positive immunostaining only in NEB cell nuclei that were Mash1-negative (Figure 2B), indicating cell-specific expression of these neurogenic markers of cell differentiation and maturation. Immunostaining for HIF-1alpha of the same NEB as in Figure 2A and B showed few positive nuclei; some coexpressed Mash1, while others expressed HIF-1alpha alone (Figure 2C). This was confirmed by triple immunostaining and a merged image of the same NEB, which show coexpression of Mash1 and HIF-1alpha in some NEB nuclei but not others (Figure 2D).

**Figure 3:**
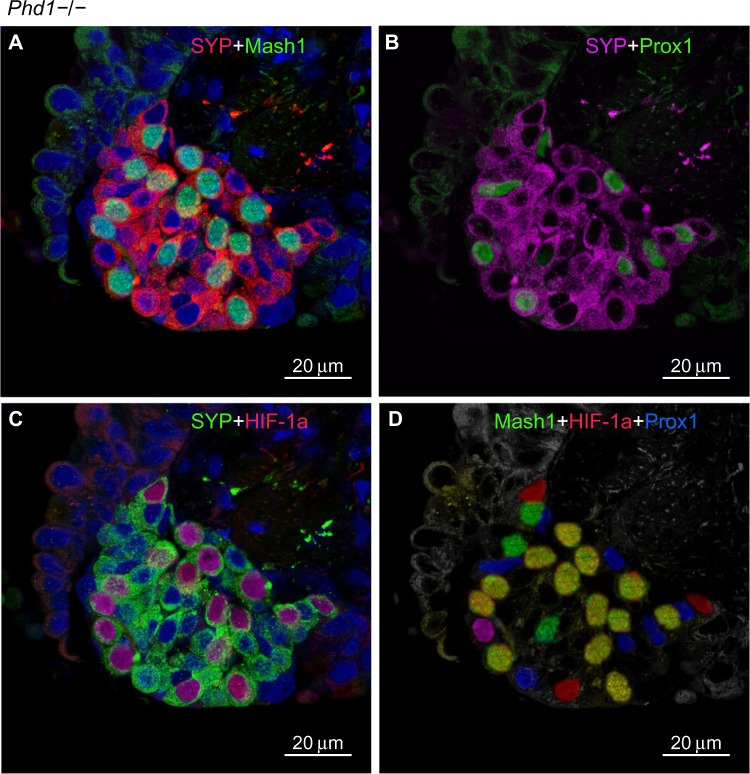
Hyperplastic neuroepithelial bodies (NEBs) with up to 40 nuclei in *Phd1*−/− mouse lungs. **Notes:** (**A**) Double immunostaining for synaptophysin (SYP) (rhodamine, red) and Mash1 (FITC, green). The majority of nuclei are Mash1-positive. (**B**) Same NEB as in (**A**), after antigen stripping and re-probing using anti-Prox1 antibody. Prox1 is localized to Mash1-negative nuclei (scale bar 20 μm). (**C**) Double immunostaining for SYP and hypoxia-inducible factor (HIF)-1alpha in the same NEB as in (**A**). Most HIF-1alpha-positive nuclei colocalize with Mash1 (compare with [**A**]). (**D**) Merged image of triple immunostaining for Mash1, HIF-1alpha, and Prox1. Up to 13 nuclei coexpress Mash1 and HIF-1alpha, while the remainder express a single epitope of Mash1, HIF-1alpha, or Prox1. **Abbreviation:** FITC, fluorescein isothiocyanate.

In agreement with our previous study of *Phd1*−/− mice, there was striking hyperplasia of NEBs compared to WT controls^10^ (Figure 3; Table 2). The mean frequency of NEB (% SYP) was significantly increased (2.86±0.8) compared to WT controls (0.74±0.2; *P*<0.01), as was the size of NEBs (2,505±406 vs 748±227; *P*<0.01) (Table 2; Figure 1). The mean number and size of NEB cell nuclei were also significantly increased, consistent with both NEB hyperplasia and hypertrophy (Table 2; Figure 1). Some NEBs in *Phd1*−/− mice contained up to 40 nucleated cells (Figure 3A–D). In terms of Mash1 expression, up to 90% of NEB cell nuclei were positive, suggesting enhanced differentiation from precursor cells (Figure 3A; Table 2). In contrast, immunostaining for Prox1, a marker of NEB maturation, revealed that only a small proportion of, mostly Mash1-negative, nuclei were expressing Prox1 (Figure 3B). Immunostaining for HIF-1alpha was comparable to Mash1 with up to 72% of NEB cell nuclei expressing HIF-1alpha, and up to 54% coexpressing both Mash1 and HIF-1alpha, suggestive of a hypoxia or HIF-driven process (Figure 3C). Triple immunostaining and merged imaging confirmed high levels of Mash1 and HIF-1alpha coexpression and the absence of coexpression of Prox1 with HIF-1alpha, suggesting that NEB cell differentiation, but not their maturation, is HIF-1alpha-dependent (Figure 3D).

**Figure 4:**
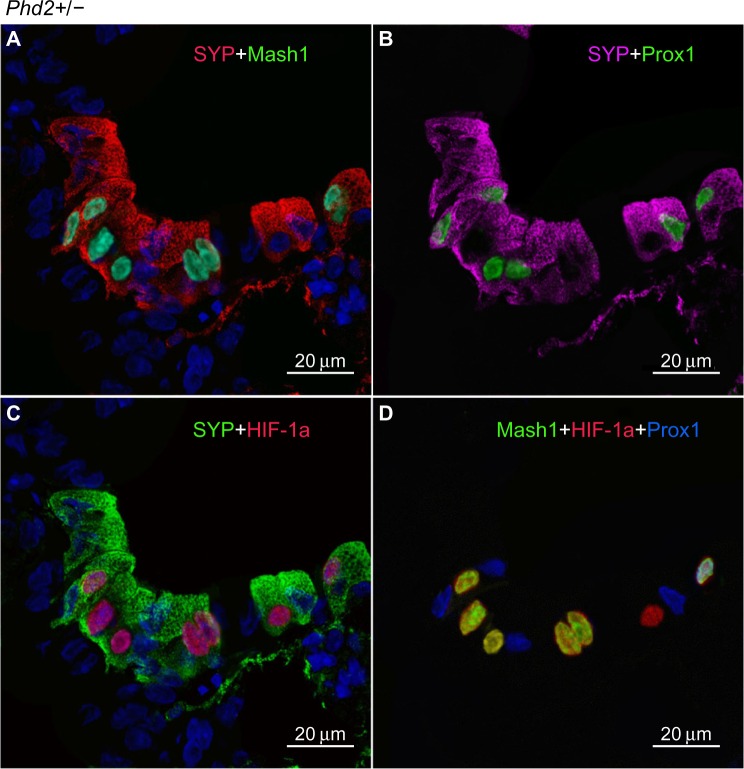
Moderately hyperplastic neuroepithelial bodies (NEBs) in the lungs of *Phd2*+/− mice. **Notes:** (**A**) Synaptophysin (SYP) and Mash1 immunostaining is comparable to those in *Phd1*−/− mice but with fewer nuclei. (**B**) Prox1 is localized to Mash1-negative nuclei (compare with [**A**]) (scale bar 20 μm). (**C**) Double immunostaining for SYP and hypoxia-inducible factor (HIF)-1alpha in the same NEB as in (**A**). Most HIF-1alpha-positive nuclei colocalize with Mash1 (compare with [**A**]). (**D**) Merged image of triple immunostaining for Mash1, HIF-1alpha, and Prox1. Five nuclei coexpress Mash1 and HIF-1alpha while the remainder express a single Mash1, HIF-1alpha, or Prox1 epitope.

In *Phd2*+/− mice, the distribution and number of NEBs (% SYP) were comparable to WT controls (Table 2). However, the NEBs showed evidence of hypertrophy as their size/area were moderately increased with statistically significant differences from WT controls (*P*<0.05), indicating an effect of PHD2 even in the heterozygous state (Figures 1 and 4; Table 2). On the other hand, there was no evidence of NEB hyperplasia, since the number and size of NEB cell nuclei were comparable to WT controls (Table 2; Figure 1). While the % of Mash1 and Prox1-positive nuclei was similar to WT controls (Figure 4A and B; Table 2), the % of nuclei co expressing Mash1 and HIF-1alpha was significantly increased, possibly reflecting the early stages of a hypertrophic response (Figure 4C and D; Table 2).

**Figure 5:**
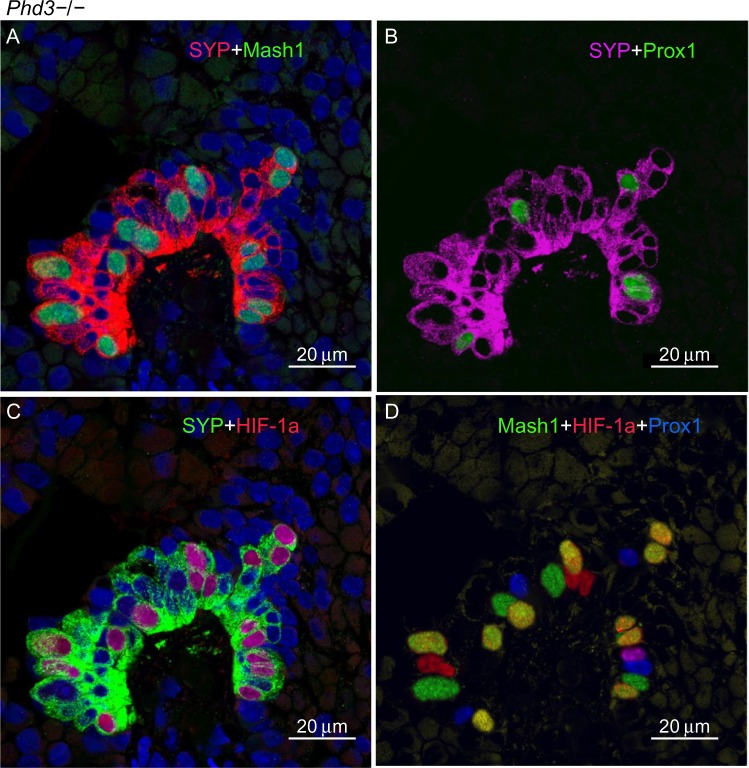
A representative neuroepithelial body (NEB) in the lungs of *Phd3*−/− mice. **Notes:** (**A**) Hyperplastic synaptophysin (SYP) immunoreactive NEB cells with many Mash1 immunoreactive nuclei. (**B**) Same NEB as in (**A**), re-probed for Prox1. Note Prox1 expression in Mash1-negative nuclei (compare with [**A**]; FITC, green) (scale bar 20 μm). (**C**) Double immunostaining for SYP and hypoxia-inducible factor (HIF)-1alpha. The majority of Mash1-positive nuclei in (**A**) also express HIF-1alpha. (**D**) Merged image of triple immunostaining for Mash1, HIF-1alpha, and Prox1. Up to ten nuclei coexpress Mash1 and HIF-1alpha while the remainder express a single epitope of Mash1, HIF-1alpha, or Prox1. This expression pattern is similar to that observed in NEBs in *Phd1*−/− mice. **Abbreviation:** FITC, fluorescein isothiocyanate.

In contrast, *Phd3*−/− mice showed striking NEB hyperplasia and hypertrophy comparable to that of the *Phd1*−/− mice noted earlier (Figures 1 and 5; Table 2). In *Phd3*−/− mice, the mean % of SYP-positive NEB area was more than double that of WT controls (2.10±0.76 vs 0.89±0.4, *P*<0.01). Similarly, NEB size and the number and size of NEB cell nuclei were significantly increased in *Phd3*−/− mice compared to WT controls, and was comparable to the *Phd1*−/− genotype (Figures 1 and 5; Table 2). The values for % of Mash1, Prox1, and HIF-1alpha-positive nuclei were comparable to those of *Phd1*−/− mice (Figure 5; Table 2), indicating that total deficiency of either *Phd1* or *Phd3* genes leads to significant hyperplasia and hypertrophy of NEBs.

**Figure 6:**
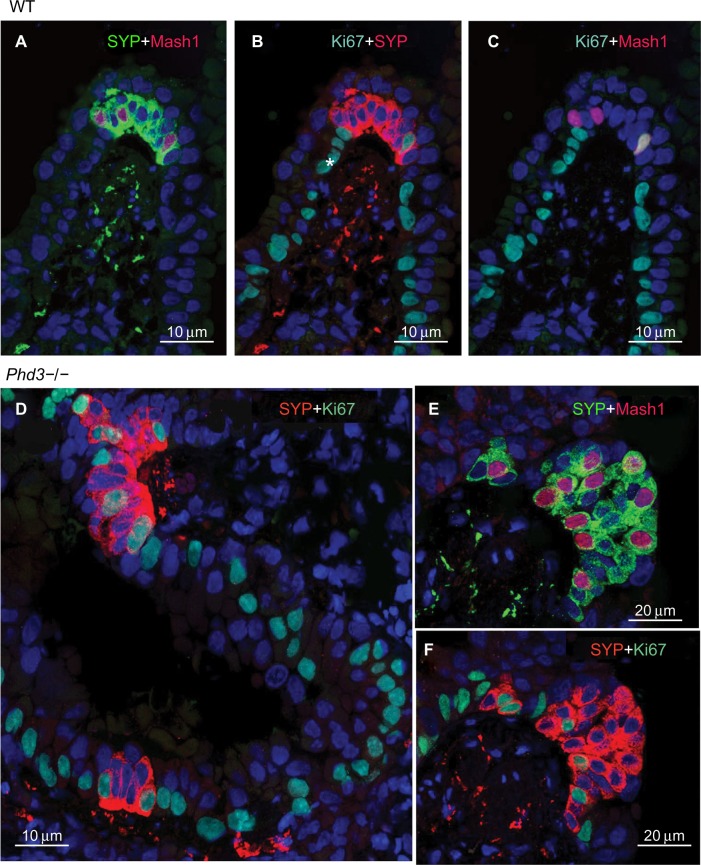
Multiple immunolabeling for the proliferation marker Ki67 together with synaptophysin (SYP) and Mash1 in wild-type control (**A**–**C**) and *Phd3*−/− mice (**D**–**F**). **Notes:** (**A**) A neuroepithelial body (NEB) at an airway bifurcation immunoreactive for SYP in cytoplasm and Mash1 in a few nuclei. (**B**) Immunostaining for Ki67 combined with SYP showing the same NEB as in (**A**). Note diffuse immunolabeling of basal cells of airway epithelium (asterisk) (FITC, green). Only a single NEB cell (far right) is Ki67-positive. (**C**) Merged image showing coexpression of Mash1 and Ki67 in a single NEB nucleus (far right) (scale bar 10 μm). (**D**) Double immunostaining for SYP and Ki67 in the lungs of *Phd3*−/− mice. A hyperplastic SYP-immunoreactive NEB with about half of nuclei immunoreactive for Ki67, suggesting cell division (scale bar 10 μm). (**E**) Double immunostaining for SYP and Mash1 in another hyperplastic NEB. Most NEB cell nuclei express Mash1 (scale bar 20 μm). (**F**) Same NEB as in (**E**) after antigen stripping and re-probing for Ki67. Only a few NEB cells express Ki67 confined to Mash1-negative nuclei. **Abbreviations:** WT, wild-type; FITC, fluorescein isothiocyanate.

Immunolabeling for the cell proliferation marker Ki67 in all *Phd*-deficient mice, as well as WT controls, showed almost uniform labeling of airway epithelial basal cells, in keeping with their high turnover rate (Figure 6; Table 2). Only very occasional Ki67-positive nuclei were seen in NEB cells, reflecting their low doubling time.^24^ However, quantitative assessment revealed that Ki67 labeling of NEBs in *Phd1*−/− and *Phd3*−/− mice was significantly higher when compared to WT controls (Figure 6; Table 2). In *Phd2*+/− mice, the Ki67 labeling was increased approximately twofold (Table 2). NEBs in both WT and *Phd*-deficient mice showed only very occasional nuclei coexpressing both Ki67 and Mash1 (Figure 6). These findings suggest that the NEB hyperplastic response is at least partially mediated by increased cell division.

## Discussion

The present study examined the effects of inactivation of different PHD isoforms on the distribution, frequency, and size of NEBs, the presumed airway O_2_ sensors. We confirm our earlier observations that NEBs in *Phd1*−/− mice undergo marked hyperplasia and hypertrophy. In addition, we also show a similar degree and extent of NEB hyperplasia/hypertrophy in *Phd3*−/− mice, while only moderate changes were observed in *Phd2*+/− mice, suggesting that partial deficiency is insufficient to induce a full NEB cell hyperplasia/hypertrophy response. Hyperplasia and hypertrophy are general terms that describe increased cell number and cell size, respectively. An increased size of the nuclei implies increased DNA synthesis in cells with low mitotic activity. Although hyperplasia and hypertrophy are two distinct processes, they frequently occur together and may be triggered by the same stimuli. For example, chronic hypoxia is a well-known stimulus to induce hyperplasia/hypertrophy in hypoxia sensing neurosecretory cells, such as CB glomus cells, which is often associated with altered chemoreceptor function.^25^ Hyperplasia of NEBs has been reported in individuals living at high altitude and animal models exposed to chronic hypoxia.^9^ However, the mechanisms of hypoxia-induced NEB hyperplasia are currently unknown.

It should be noted that acute (milliseconds to seconds) O_2_ sensing by PNEC/NEB cells relies on a number of preformed proteins, including NADPH oxidase or NADPH oxidase 2, composed of a catalytic membrane subunit, a heterodimer containing gp91^phox^ and p22^phox^, and cytoplasmic regulatory subunits p47^phox^, p67^phox^, and Rac2, which constitute the functional NADPH oxidase 2.^7^,^26^ The assembled complex generates a superoxide that is rapidly converted to H_2_O_2_ by superoxide dismutase. In turn, H_2_O_2_ is used as a messenger molecule gating K^+^(O_2_) channels via an amino terminus cysteine residue, which is known to be highly redox sensitive.^7^,^26^,^27^ This acute O_2_ sensing mechanism appears to be unique to PNEC/NEBs; in other O_2_ neurosecretory cells (ie, CB glomus cells, adrenal medullary cells), alternate mechanisms are involved.^5^ In contrast, sensing of chronic/sustained hypoxia (hours to days) involves diverse transcriptional responses that share a common O_2_ sensing process mediated by the post translational hydroxylation of specific prolyl residues in HIFs.^18^,^28^ Under conditions of reduced O_2_ availability, HIF induces the expression of genes that mediate a broad range of adaptive responses. HIF prolyl hydroxylation is catalyzed by PHD domain enzymes that act as cellular O_2_ sensors though obligate use of O_2_ in the hydroxylation reaction. Functionally different PHD isoforms may contribute differentially to specific physiological processes, although this remains poorly understood.^12^,^13^,^28^,^29^ PHD2 is the most abundant PHD enzyme expressed in most tissues and also the most important in setting normoxic levels of HIF-1alpha.^29^,^30^ In contrast, PHD1 and PHD3 are usually expressed at lower levels and make greater contribution to the regulation of HIF-2alpha.^29^ While *Phd1*−/− and *Phd3*−/− mice are viable, the *Phd2*−/− genotype is embryonically lethal.^31^ Hyperplasia has been noted in sympathoadrenal tissues of *Phd2*+/− and *Phd3*−/− mice, with CB hyperplasia in *Phd2*+/−21,32 and *Phd3*−/−20 mice and an increased number of cells in the superior cervical ganglion and adrenal medulla of *Phd3*−/− mice.^20^ Inactivation of *Phd1*, on the other hand, has been associated with hypoplasia rather than hyperplasia in other tissues with hypoproliferation in breast tissues of older lactating *Phd1*−/− mice.^33^ It is not yet clear whether all these effects are mediated through the regulation of HIF as a number of alternative non-HIF substrates have been described for particular PHD isoforms. In the current work, we have not formally demonstrated that the observed hypertrophic/hyperplastic responses are mediated by HIF, but associated staining for HIF-1alpha and effects following inactivation of the different *Phd*s would support this.

This is the first report of detailed morphometric analysis of PNEC/NEB cells in genetic models of *Phd* deficiency. We show that the inactivation of either *Phd1* or *Phd3* leads to significant hyperplasia and hypertrophy of NEB cells suggesting a nonredundant function since a deficiency of either isoform does not lead to compensation by the other. The NEB hyperplasia was manifest as an increase in the number of NEB cells, whereas the hypertrophy was manifest as an increase in the size of the NEBs as well as the increased size and number of NEB nuclei in *Phd1*−/− and *Phd3*−/− mice. These hyperplastic/hypertrophic effects are reminiscent of the effects of chronic hypoxia on NEBs in vivo and in vitro.^9^ The use of our novel method of multilabel immunofluorescence on formalin-fixed, paraffin-embedded sections permitted visualization of both cytoplasmic and nuclear epitopes in the same cells and thus was instrumental for the assessment of potential mechanisms of NEB cell hyperplasia.^14^ Our findings suggest that differentiation from precursor cells is the predominant mechanism for NEB cell hyperplasia as documented by increased expression of Mash1 gene, a critical transcription factor for PNEC/NEB cell differentiation.^15^ Developmental studies have shown that in fetal lung, Mash1 (or achete-scute homolog-1, hASH1, in humans) expression precedes PNEC/NEB cell differentiation and acts in concert with Hes1, a transcription factor involved in differentiation of other airway epithelial cells.^16^,^34^ In addition to its role in normal lung development, Mash1 (or hASH1) has been implicated in a variety of pulmonary neoplasms.^34^ PNEC is the first cell type to differentiate within the primitive airway epithelium via differentiation from precursor cells and seldom by mitosis.^24^ In a normal postnatal lung, only very few or no PNEC/NEBs show Mash1 expression, although we have previously shown that hypoxia modulates PNEC/NEB cell differentiation during early fetal lung development via Mash1 expression.^17^

Our findings may be relevant to recent investigations of PNEC/NEB cell hyperplasia in several pediatric lung disorders, including chronic lung disease of prematurity (bronchopulmonary dysplasia), sudden infant death syndrome, and neuroendocrine cell hyperplasia of infancy.^2^,^35^ These conditions are all characterized clinically by chronic hypoxia and abnormal breathing, suggesting a causative relationship to PNEC/NEB hyperplasia. Our recent studies of these conditions using formalin-fixed, paraffin-embedded sections and multilabel immunohistochemistry, as applied here, revealed that the molecular signature of PNEC/NEB cell hyperplasia in these human diseases closely resembles that of mice models of genetic ablation of *Phd*s.^35^ Since some cases of bronchopulmonary dysplasia, sudden infant death syndrome, and neuroendocrine cell hyperplasia of infancy appear to be familial, the possibility of a genetic defect has been postulated. Recent studies reported germline mutations in the *Phd1* and *Phd2* genes in association with systemic hypertension and pheochromocytoma, paraganglioma–polycythemia syndrome.^36^ The possibility of defects in *Phd* genes in the aforementioned pediatric lung disorders has not yet been explored. The relevance of our studies may extend to adults since pulmonary neuroendocrine neoplasms are commonly derived from PNEC/NEB cells and hypoxia sensing mechanisms are likely involved in the pathobiology of these tumors as they possess O_2_ sensing properties similar to their normal counterparts.^37^,^38^ Further cellular, molecular, and genetic studies to fully define the precise mechanisms of PNEC/NEB hyperplasia and their physiological consequences in both animal models and humans will be important as these conditions are associated with high morbidity and mortality with little effective therapy.

## Figures and Tables

**Table 1:** Antibody types, dilutions used, and sources

Primary antibodies	Dilutions	Sources
Synaptophysin	1:100	BD Biosciences (San Jose, CA, USA)
Ms anti-Mash1 mAb	1:100	Bio-Rad Laboratories (Hercules, CA, USA)
Rb anti-Prox1 pAb	1:100	Abcam (Cambridge, UK)
Rb anti-Ki67 pAb	1:100	Abcam
Rb anti-HIF-1alpha	1:100	Abcam
**Secondary antibodies**	**Dilutions**	**Sources**
Anti-rabbit IgG ALEXA 568	1:500	Thermo Fisher Scientific (Waltham, MA, USA)
Anti-mouse IgG ALEXA 488	1:500	Thermo Fisher Scientific
Anti-mouse IgG H+L-biotin	1:200	Jackson ImmunoResearch (West Grove, USA)
Anti-rabbit IgG H+L-biotin	1:200	Jackson ImmunoResearch
Anti-rabbit IgG H+L-Rhodamine	1:100	Jackson ImmunoResearch
Anti-rabbit IgG H+L-FITC	1:200	Jackson ImmunoResearch
Anti-rabbit IgG ALEXA 680	1:200	Jackson ImmunoResearch
Anti-mouse IgG H+L-Texas Red	1:1,000	Jackson ImmunoResearch
Streptavidin-Texas Red X	1:1,000	Thermo Fisher Scientific
RedDot-2 dye (697 nm)		Biotium (Hayward, CA, USA)

**Abbreviation:** FITC, fluorescein isothiocyanate.

**Table 2:** Morphometric analysis of NEBs in mouse lungs in three *Phd* genotypes

Genotypes	n	% IMS^1^	NEB size^2^	Nuclear no^3^	Nuclear size^4^	M-P no^5^	H-P no^6^	Co-S no^7^	PX1-%^8^	Ki67-%^9^
*Phd1*−/−	4	2.86±0.8	2,505±406	112±19	208±38	90±20	72±21	54±18	9.0±4.3	12±4
WT	4	0.74±0.2	748±227	38±9	104±41	5.8±1.7	1.2±0.4	1.0±0.1	25±7.1	2.6±0.7
*Phd2+/*−	4	0.78±0.2	1,049±174	48±14	112±21	4.6±1.2	0.9±0.2	6.2±0.2	6.2±2.0	4.8±0.4
WT	4	0.72±0.2	865±162	41±8.4	109±24	4.9±0.5	1.2±0.4	1.5±0.2	29.4±0.2	1.5±0.4
*Phd3*−/−	4	2.10±0.76	1,941±509	107±19	209±52	84±17	66±17	52±16	9.2±2.8	8.8±5.0
WT	4	0.89±0.4	878.2±199	36±11	129±33	6.1±1.8	1.1±0.1	0.4±0.1	30.1±6.1	0.7±0.1

**Note:**

1 % IMS ± SEM: percent of immunostaining area per 100 mm^2^ airway epithelia;

2 NEB size in integrated area of positive immunostaining;

3 Total number of nuclei in NEB;

4 Mean nuclear size of NEBs in integrated area;

5 Mean Mash1-positive cells;

6 HIF-1α-positive cells;

7 Costaining Mash1 and HIF-1α;

8 Percentage of Prox-1-positive cells in NEBs;

9 Percentage of Ki67-positive cells in NEBs.

**Abbreviations:** HIF, hypoxia-inducible factor; IMS, immunoreactive staining; NEB, neuroepithelial body; SEM, standard error of the mean; WT, wild-type.
